# AD|ARC (Administrative Data Agri-Research Collection): Linking Individual, Household and Farm Business Data for Sociodemographic Research on Farming Households

**DOI:** 10.23889/ijpds.v9i5.2835

**Published:** 2024-09-18

**Authors:** Sian Morrison-Rees, Laura Madden, Matthew Curds, Paul Caskie, Rachael Loftus

**Affiliations:** 1 Swansea University; 2 Welsh Government; 3 Agri-Food and BioSciences Institute (AFBI)

## Objective

To understand the socio-demographic characteristics and structure of farming households in Wales who receive subsidies compared to non-farming rural households.

## Approach

We utilised the AD|ARC dataset, a comprehensive resource we created within the SAIL databank by linking de-identified records of farms receiving farming subsidies in Wales with socio-demographic information from the Census 2011. A control group was created by matching each farming household with up to three households in the 2011 Census from a similar geographical area that did not contain a farmer or household member in receipt of farming subsidies. We used Census 2011 variables to compare household size, family structure and age for 18,135 farming households with a control group of 53,365 rural non-farming households.

## Results

We found little difference between the age and gender distribution between cohorts, however we found significant differences in the household structure. Farming households had a larger average size compared to non-farming households. Single-occupancy farming households had a significantly higher proportion of males, farming households were more likely to have couples with children and a higher percentage of these couples were married compared to non-farming households.

## Implications

The agriculture sector in Wales is facing a range of challenges linked to social, economic, and environmental sustainability as it establishes a new direction after UK withdrawal from the EU. Understanding the sociodemographic characteristics of farming communities is essential for developing effective policies and interventions that address the specific needs and challenges faced by these groups.

